# Long-term morphologic fundus and optic nerve head pattern of progressive myopia in congenital glaucoma distinguished by age at first surgery

**DOI:** 10.1038/s41598-020-67051-0

**Published:** 2020-06-22

**Authors:** Eun Jung Lee, Jong Chul Han, Do Young Park, Changwon Kee

**Affiliations:** Department of Ophthalmology, Samsung Medical Center, Sungkyunkwan University School of Medicine, Seoul, Korea

**Keywords:** Optic nerve diseases, Refractive errors

## Abstract

The purpose of this study was to investigate the preservation of round optic nerve head (ONH) shape in myopic eyes of surgically treated congenital glaucoma patients, with regard to factors associated with intraocular pressure (IOP) elevation-induced peripapillary scleral (PPS) deformation. Using optical coherence tomography (OCT) on the ONH and macula, we identified myopic eyes with round ONH and internally oblique border tissue and those with non-round ONH. We investigated differences in clinical factors between the two groups. We included 51 eyes of 34 patients. Age at first surgery (2.8 vs. 15.2 months, P < 0.001) was significantly different between the two groups. Axial length was also significantly longer (P = 0.004) in the non-round group, but multiple logistic regression analysis revealed age as the only significant factor (P < 0.05) in ONH roundness. Interestingly, the round ONH group also had non-curved fundus morphology and a thick choroid, while the non-round ONH group showed diverse degrees of disc tilt and posterior pole curvature, and a thin choroid. In conclusion, in eyes with congenital glaucoma, age at first surgery, particularly when older than 6 months, was associated with round ONH and emmetropia-like fundus despite high myopia. The findings may indicate two different changes in the posterior sclera and the neural canal in response to the increased IOP.

## Introduction

Tilted optic discs and myopia are significantly associated, as shown in many large-scale studies^1–6^. In congenital glaucoma, myopia is common in 50–70% of patients^7–10^. Therefore, one might expect a tilted disc in congenital glaucoma eyes with marked axial myopia. However, the optic nerve head (ONH) and optic disc morphology have not been deeply investigated in congenital glaucoma patients, especially whether disc tilting is present. It has rarely been reported that the optic discs of myopic congenital glaucomatous eyes are round, not tilted, except in a single report^11^.

Indeed, the optic discs of healthy myopic eyes can be free of tilt^1,12,13^, and the round discs in myopic congenital glaucoma eyes may be insignificant variants. However, for the eyes that are distinguished by a period of significant intraocular pressure (IOP) elevation compared to normal myopic eyes, the round optic disc shape may be a result of strain in the axially elongated eyeball.

This concept is supported by the biomechanical geometry of the scleral shell around the neural canal opening. It is important to note that the collagenous tissues not only elongate, but also stiffen in response to mechanical forces^14,15^; such tissues include the sclera^16,17^. The stiffening is more pronounced in the circumferentially reinforced peripapillary sclera (PPS) region, as reported in the studies on human eyes^18^, animals^19,20^, and theoretical modelling^21^. It is the circular arrangement of the collagen fibres that correlate this stiffening effect with the round PPS structure around the optic disc.

We may have been biased because of the high elasticity of young eyes as reflected by the general eyeball enlargement^22,23^. All coats including Bruch’s membrane (BM), the choroid, and the sclera, are thought to be stretched and extended as in an expanding air balloon, especially within the first 2 years of life^24^. However, the round-fashioned scleral stiffening with loading enables a response other than uniform “expansion” of the posterior sclera.

In this study, we aimed to identify biologic parameters associated with optic disc roundness in a group of surgically treated myopic congenital glaucoma patients. We investigated the optic disc shape and clinical factors and performed OCT imaging on the ONH and the macula. A subset of patients avoided progressive optic disc tilting despite a substantial axial length increase. The age at the first surgery broadly determined the “fixed” roundness of ONH, while other results suggested more complex underlying mechanisms beyond the age difference.

## Results

We included 51 eyes of 34 patients with myopic congenital glaucoma. Of these, 20 patients (58.8%) were male and 22 (64.7%) had bilateral glaucoma. Patients received their first surgery at 8.4 ± 8.8 (range, 0.2–34.1) months and had been followed up for 13.3 ± 4.9 (4.6–21.2) years. Age at the most recent visit was 14.8 ± 5.5 (5.3–25.6) years. The final axial length was 28.61 ± 2.62 (25.16–36.85) mm and 45 eyes (90.2%) had high myopia with axial lengths > 26.0 mm.

**Figure 1:**
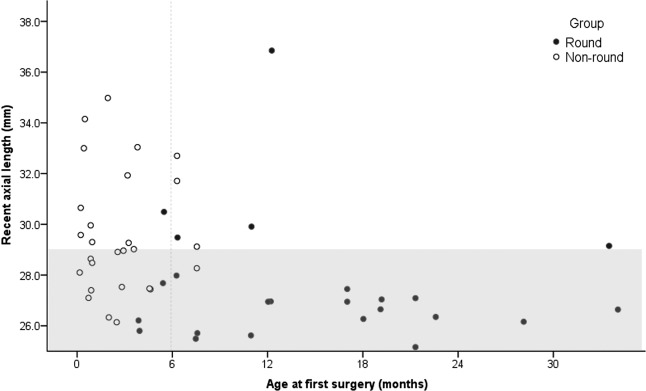
Distribution of axial length and age at first surgery in the round and non-round groups of myopic congenital glaucoma patients. The graph shows the relationship between age at first surgery and axial length in two groups. The round group (black filled dots) showed a shorter axial length than the non-round group (black un-filled dots), but the effect of age on grouping is also evident. An arbitrary grey line splitting the distribution between the two groups is marked at 6 months. The shade indicates the axial length from 25.0 to 29.0 mm.

Table 1 shows the comparative data. Age at first surgery was significantly different between the groups; the non-round group showed a significantly earlier age at first surgery, starting from 5 days after birth and ranging up to 6–7 months in most cases. Little overlap was observed between the ages in the two groups, and the distribution was almost bipartite (Fig. 1). Axial length was also significantly longer in the non-round group than in the round group (P = 0.004). When separately analysed for the eyes with recent axial length between 25.0 and 29.0 mm, the results were still significant (P = 0.018). Each group showed a high percentage of high myopia of over 80%.

**Table 1:** Comparison between the Non-round and Round Groups in Myopic Congenital Glaucoma.

Parameter	Non-round group	Round group	P value*
Number of eyes	28	23	
Ovality index	1.489 ± 0.224 (1.111–1.944)	1.042 ± 0.024 (1.011–1.085)	0.036
Demographics			
Sex (female/male)	15/13	7/16	0.206
First surgery age (months)	2.8 ± 2.4 (0.16–7.57)	15.2 ± 8.9 (3.87–34.07)	<0.001
First surgery age for the eyes with recent axial length between 25.0 and 29.0 mm (months)	2.5 ± 2.1 (0.16–7.54)	15.1 ± 8.5 (3.87–34.07)	0.018
Preoperative findings			
IOP (mmHg)	31.4 ± 7.1	28.7 ± 4.2	0.134
Follow-up biometrics			
Recent axial length (mm)	29.5 ± 2.5	27.1 ± 1.4	0.004
High myopia (>26.0 mm), %	96.4	82.6	0.132
Axial length around 10 years (mm)	28.9 ± 2.5	26.9 ± 1.4	0.030
PPS-ONH OCT parameters			
BM without RPE (zone β)	266.0 ± 215.2	28.8 ± 51.2	0.002
Scleral border tissue length (zone γ) (µm)	828.4 ± 824.1	22.2 ± 48.8	<0.001
Horizontal BMO diameter (µm)	2258.0 ± 943.3	1631.4 ± 264.7	0.013
Vertical BMO diameter (µm)	2176.4 ± 505.9	1661.3 ± 246.8	0.006
Average BMO diameter (µm)	2187.2 ± 604.7	1641.6 ± 205.3	0.003
Ratio of H/V BMO diameter	0.985 ± 0.194	0.982 ± 0.061	0.933
Macular BM distance (µm)	3906.4 ± 791.6	3860.0 ± 313.0	0.811
Mean LC depth (µm)	565.9 ± 225.7	602.6 ± 194.6	0.539
Macular BM curvature angle (°)	2.78 ± 0.2.60	−0.41 ± 0.92	0.039
Subfoveal choroidal thickness (µm)	184.9 ± 107.7	322.9 ± 89.6	0.002
Follow-up clinical parameters			
IOP at OCT measurements (mmHg)	14.9 ± 3.38	16.0 ± 3.92	0.437
Follow-up period (years)	12.2 ± 4.2	14.6 ± 5.4	0.128
Total number of surgeries (times)	2.81 ± 1.76	2.13 ± 0.99	0.124
Age at the last surgery (years)	3.44 ± 4.67 (0.01–19.06)	4.98 ± 4.70 (0.45–15.70)	0.394
Final BCVA (LogMAR)^†^	0.798 ± 0.797	0.633 ± 0.637	0.479

Values are mean ± standard deviation (range) unless otherwise specified.

IOP = intraocular pressure, PPS = peripapillary sclera, ONH = optic nerve head, OCT = optical coherence tomography, BM = Bruch’s membrane, RPE = retinal pigment epithelium, BMO = Bruch’s membrane opening, H/V = horizontal/vertical, LC = lamina cribrosa, BCVA = best corrected visual acuity, LogMAR = logarithm of the Minimum Angle of Resolution.

*P value was calculated using generalised estimating equation analysis.

^†^The arbitrary LogMAR values for visual acuity less than counting fingers were used as follows: counting fingers were converted to 2.0 LogMAR units, hand motions were converted to 2.3 LogMAR units, light perception was converted to 2.5 LogMAR units, and no light perception was converted to 3.0 LogMAR units^68^.

There was no difference regarding the total number of surgeries or the age at which the last surgery was performed. Moreover, 72.5% were distributed within the age of 4.64 years; 37.3% of the eyes received surgery only once, and 80.4% received surgery less than three times. In those eyes for which the last surgery was performed after 10 years of age, the interval between the last and the precedent surgery was considerably long, 9.16 ± 5.08 years, suggesting a minimal influence on the optic disc shape that had already been established. For example, in those eyes for which the last surgery was performed at 19.06 and 15.70 years of age, the precedent surgery had been performed 18.59 and 15.37 years earlier, respectively.

Multiple logistic regression revealed that the only significant parameter was the age at first surgery (Table 2). To compensate for the various axial length measurement ages, we chose only eyes with axial length measurements within the specific age range of 9–13 years. Thirty-three eyes (64.7%) – 19 eyes in the non-round group and 14 eyes in the round group – fulfilled the age range criteria, with an average age of 10.9 ± 1.1 (9.1–13.4) years. No significant difference was observed in the measurement age between the round and non-round groups (P = 0.389).

**Table 2:** Multiple Binary Logistic Regression with Preservation of the ONH Roundness as the Primary Outcome.

	Univariable analysis			Multivariable analysis model 1			Multivariable analysis model 2		
	Odds ratio	95% CI	P value^*^	Odds ratio	95% CI	P value^*^	Odds ratio	95% CI	P value^*^
Sex	2.637	0.586–11.867	0.206						
First surgery age	1.020	1.009–1.031	**<0.001**	1.022	1.008–1.036	**0.003**	1.019	1.002–1.038	**0.033**
Preoperative IOP	0.921	0.827–1.026	0.134						
Recent axial length	0.512	0.326–0.803	**0.004**						
Axial length around 10 years^†^	0.684	0.485–0.964	**0.030**	0.433	0.186–1.006	0.052	0.730	0.415–1.284	0.275
Follow-up period	1.000	0.999–1.001	0.128						
Average BMO diameter	0.994	0.990–0.998	**0.003**				0.994	0.986–1.002	0.142

CI = confidence interval, PPS = peripapillary sclera, ONH = optic nerve head, IOP = intraocular pressure.

^*^P value was calculated using binary logistic generalised estimating equation analysis.

^†^Axial length around 10 years of age was used. Measurement was performed at 11.0 ± 1.3 (9.3–13.4) years and 10.7 ± 0.7 (9.1–12.0) years in the non-round and round groups, respectively, without significant differences between the two groups (P = 0.389). Thirty-three eyes were included in the analysis.

### Interval change of ovality index and axial length

We observed that the preserved roundness of ONH started even from the youngest ages (Table 3). The round group showed an ovality index near 1.0 from the earliest documentation, which included two eyes in patients under 6 months of age and seven eyes before the first surgery. Moreover, the ovality index had decreased during the follow-up (−0.01 on average). On the contrary, the non-round group showed an ovality index that was significantly larger than that of the round group (1.47 vs. 1.05, P = 0.004), and demonstrated an increase in the ovality index during the follow-up. In particular, the ONH of four eyes in the non-round group changed from a round to tilted shape, with the ovality index increasing from under 1.2 to over 1.5.

**Table 3:** Earliest Recordings of the Optic Disc Ovality and Axial Length.

Parameter	Non-round group	Round group	P value^*^
Number of eyes	24	23	
**Ovality index**			
Earliest optic disc ovality index	1.47 ± 0.25 (1.00–1.80)	1.05 ± 0.11 (1.00–1.40)	0.004
Recent ovality index	1.52 ± 0.19 (1.17–1.91)	1.04 ± 0.03 (1.00–1.09)	<0.001
Ovality index change	0.05 ± 0.21 (−0.35–0.49)	−0.01 ± 0.10 (−0.34–0.09)	0.224
Age at the earliest optic disc photograph (years)	1.91 ± 1.65 (0.16–5.33)	3.29 ± 1.78 (0.10–6.42)	0.056
Age at the recent optic disc photograph (years)	12.75 ± 4.32 (5.18–19.19)	15.67 ± 5.29 (225.18–22.48)	0.144
Interval (years)	10.84 ± 3.22 (4.89–14.87)	12.38 ± 3.84 (4.60–17.01)	0.262
**Axial length**			
Earliest axial length (mm)	24.75 ± 3.32 (19.20–29.90)	25.70 ± 2.17 (20.40–29.60)	0.357
Recent axial length (mm)	29.49 ± 2.18 (26.14–34.15)	27.79 ± 2.96 (25.49–36.85)	0.231
Age at the earliest axial length measurement (years)	3.67 ± 2.99 (0.04–8.55)	6.38 ± 3.31 (0.29–9.83)	0.054
Age at the recent axial length measurement (years)	11.86 ± 4.03 (3.96–18.58)	13.62 ± 5.87 (3.96–20.60)	0.413
Interval (years)	8.19 ± 3.16 (3.67–15.57)	7.24 ± 3.20 (3.09–11.20)	0.448
Normal infant axial length	17.4 ± 0.5 mm (0–1 month), 18.6 ± 0.5 mm (1–2 months), 18.9 ± 0.4 mm (2–6 months), 19.2 ± 0.5 mm (6–12months)^69^ 18 mm (>1 week–2.9 months), 18.7 mm (3–5.9 months), 19 mm (6–8.9 months), 19.2 (9–11.9 months)^70^		

Only the eyes with their earliest optic disc photograph or axial length measurement performed before 10 years of age were included in the table.

^*^P value was calculated using generalised estimating equation analysis between the non-round and round groups.

For specific examples, the two round group eyes with the earliest documentation of ovality index before 6 months of age showed an initial ovality index of 1.387 and 1.016, which decreased to 1.060 and 1.012 at the most recent visit, respectively. Seven eyes had photographs from before the first surgery, at age 0.10–1.77 years, and their ovality indices also ranged between 1.016 and 1.387. In the non-round group, 5 eyes with the earliest documentation of ovality index before 6 months of age showed an initial ovality index of 1.003–1.217, which changed to 1.170–1.611 in the follow-up; two eyes had photographs from before the surgery, whose ovality indices were 1.003 and 1.210 and had increased to 1.170 and 1.305, respectively.

We had three eyes in the round group with axial length measurements from before the first surgery. The axial lengths were 20.4 mm at four months of age, and 26.1 mm and 25.8 mm at 16.4 months of age. We had five eyes in the non-round group with axial length measurements from before the first surgery. The axial lengths were 19.9 and 19.2 mm at 13 days of age, 20.9 mm and 21.7 mm at 2.5 months of age, and 21.2 mm at 3.4 months of age.

**Figure 2:**
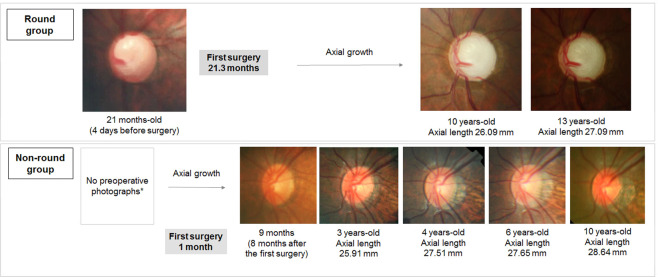
Cases with interval change of ovality index and axial length. In the round group, the ovality index is near 1.0 despite progressive axial growth. In contrast, the ovality index may show a slight increase with axial growth in the non-round group. The asterisk indicates that a preoperative photograph was unavailable for the patient due to corneal opacity.

Examples of fixed ONH roundness and increasing tilt are presented in Fig. 2 from the round group and non-round group, respectively.

### OCT findings

We obtained OCT images of high enough quality for quantification, except for 7 eyes of 5 patients − 5 eyes of 4 patients in the non-round group, and 2 eyes of 1 patient in the round group − due to poor cooperation.

**Figure 3:**
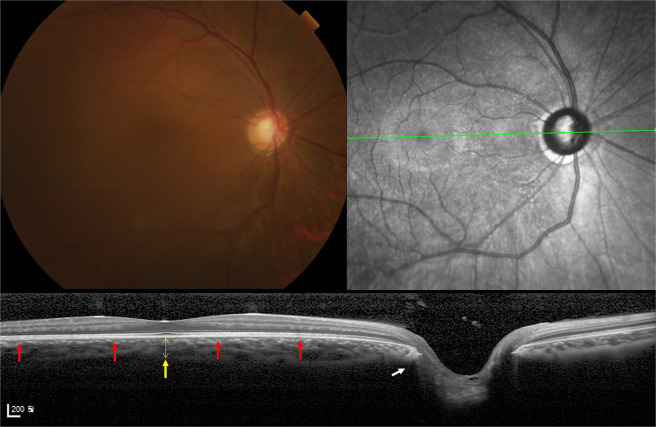
Characteristic fundus photograph and OCT of an eye in the round group. Colour fundus photograph, infrared photograph, and an OCT B-scan image connecting the fovea and centre of optic disc (FoDi) are presented. A round group eye with typical features marked with arrows: internally oblique temporal border tissue (white arrow), slightly convex Bruch’s membrane (red arrows), and thick subfoveal choroid (yellow arrow, thickness = 301 µm). Axial length was 26.99 mm. Age at first surgery was 372 days.

The OCT findings were markedly different between the two groups. The round group consistently showed the following characteristic features compared to the non-round group (Fig. 3): (1) internally oblique or minimal externally oblique border tissue (as in the definition); (2) flat or convex BM curvature; (3) thick subfoveal choroid; and (4) smaller Bruch’s membrane opening (BMO) diameter in the horizontal and vertical directions. In particular, the macular BM curvature was surprisingly emmetropia-like, being flat or slightly convex. Moreover, the round ONH morphology did not change by photographs despite the progression of myopia throughout the follow-up period.

**Figure 4:**
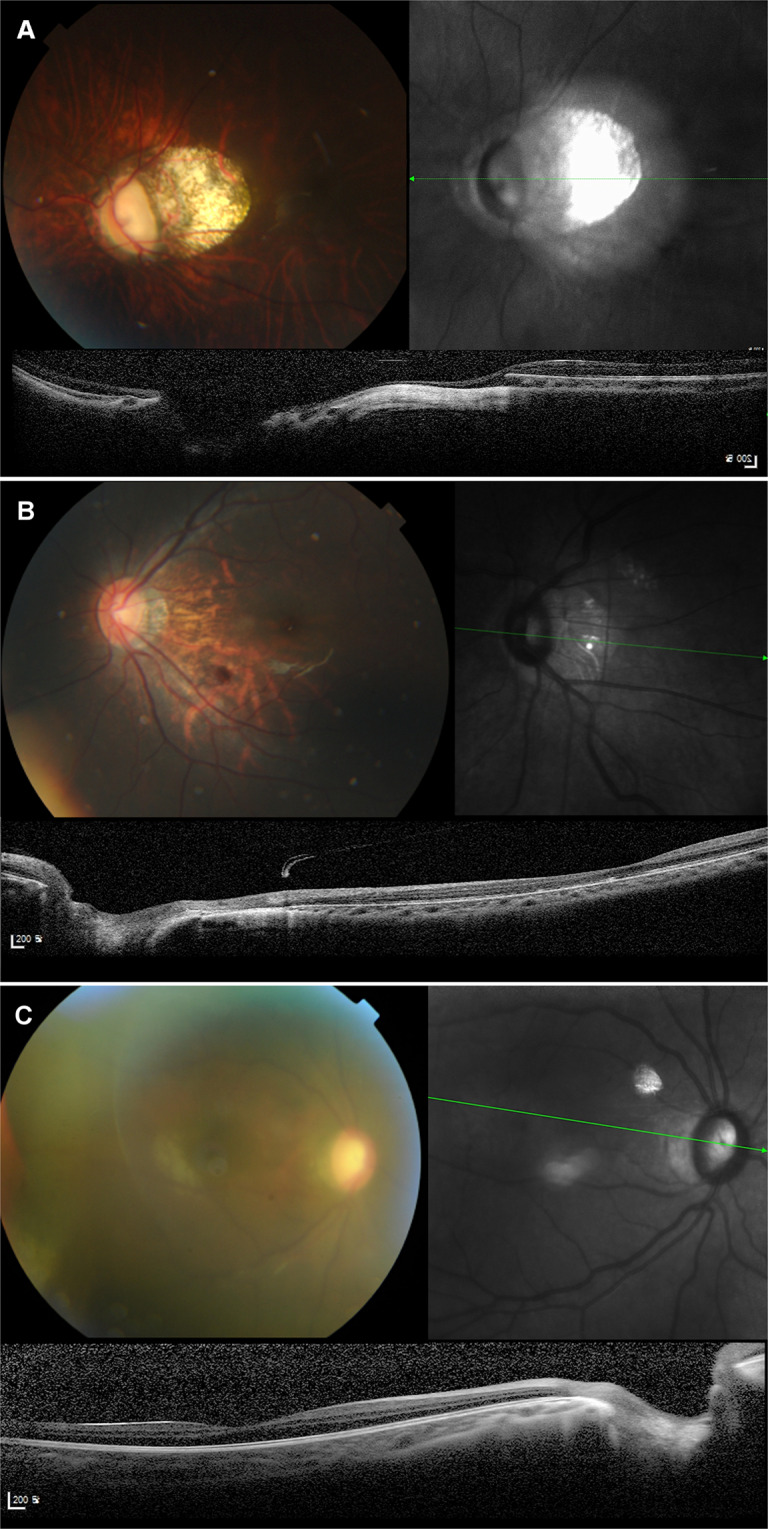
Characteristic fundus photographs and OCTs of eyes in the non-round group. Colour fundus photograph, infrared photograph, and an OCT B-scan image connecting the fovea and centre of optic disc (FoDi) are presented. (**A**) Fundus photograph shows tilted optic disc with large peripapillary atrophy and tessellated fundus. OCT shows irregular type inner scleral curvature and severely elongated externally oblique optic nerve head (ONH) border tissue. The eye had an axial length of 29.58 mm. The age at first surgery was 86 days. (**B**) Fundus photograph shows tilted optic disc with peripapillary atrophy and tessellated fundus. OCT shows sloping toward disc-type inner scleral curvature and temporally elongated externally oblique ONH border tissue. The eye had an axial length of 27.53 mm. The age at first surgery age was 26 days. (**C**) Fundus photograph shows tilted optic disc with peripapillary atrophy. OCT shows symmetric inner scleral curvature and externally oblique ONH border tissue. The eye had an axial length of 26.33 mm. The age at first surgery age was 61 days.

In contrast, the non-round group showed tilted ONHs with diverse posterior pole curvatures (Fig. 4). We classified the types of posterior pole following the suggestion by Ohno-Matsui *et al*.^25^. We observed symmetric or only minimally asymmetric (17, 60.7%), sloping toward disc (5, 17.9%), and irregular (6, 21.4%) inner scleral curvature types, while a distinct asymmetric inner scleral curvature type was absent in our group. Irregular curvature was seen in eyes with axial lengths over 29.5 mm, and sloping toward disc-type curvature was seen in eyes with axial lengths over 27.5 mm. On fundus photographs, peripapillary staphyloma was a common finding (12, 42.9%).

Due to the diversity of posterior pole shapes, we found that the calculation of macular curvature was possible only in symmetric or slightly asymmetric cases. As expected, the curvature angle was significantly larger in the non-round group than in the round group (P = 0.039), with occasional convex macular BM (Fig. 4). Interestingly, despite the tilted appearance of the optic discs, the ratio between horizontal and vertical BMOs and macular BM distance were not different between the two groups (P = 0.907 and 0.698, respectively), indicating that the BMO maintained its roundness despite severe disc tilting.

## Discussion

In congenital glaucoma, the young eyes are known to be more compliant than those of adults such that eyeball enlargement^22,26,27^, symmetric expansion of the scleral canal opening^23,28–31^, and cup reversal after IOP control^32,33^ are clinically dramatic. The immediate deformation or globe expansion due to high elasticity is thought to be sensitive only up to 2–3 years of age^22,26^.

The main conclusion of this study was that patients who received their first surgery after the age of approximately 6 months were significantly more likely to preserve the internally oblique border tissue configuration and flat macular curvature. The result may seem contrary to the common belief that ocular tissues are elastic and easily stretched in children^22,26,27^. However, it is critical to note that the general response of collagenous tissues to mechanical force is strain-stiffening^14,15^ that applies to the sclera^14,15,18–20^. Moreover, the collagens are arranged in a circumferential fashion in the PPS^18–21^. Because such stiffening would reinforce the structure of the circular network surrounding around the ONH, there may develop a resistance to distortion of the round boundary of neural canal opening despite axial growth.

The PPS stiffening in response to increased IOP is a noteworthy feature. The “structurally stiffer” PPS resists deformation and stretches less at a given elevated IOP^16^, and the PPS region in the donor eyes of the patients with glaucoma showed less deformation after increase in the IOP^18^. Scleral stiffening to increased IOP has been shown through the studies in human eyes^18^, animals^19,20^, and theoretical modelling^21^. The mechanism of stiffening involves remodelling of the extracellular matrix (ECM). Elevated IOP resulted in increased material properties in scleral tissues^16–18,34,35^. At the macroscopic level, the scleral stiffening is caused by uncrimping of the collagen fibres, thus, limiting scleral deformation under high IOP conditions^16^. Chronically, the posterior sclera becomes stiffened as a result of collagen and elastin ECM remodelling at high levels of cumulative IOP insult^16–18,36^. The scleral fibroblasts are activated in response to the mechanical strain to release matrix metalloproteinases and tissue inhibitors of metalloproteinases. Consequently they reconstruct the scleral ECM^37,38^. Other load-bearing collagenous tissues also show cellular mechanotransduction and ECM remodelling in response to increased strain^39^. Experimental stiffening of the sclera has been supported by consistent results seen both in animal models^16,34,35^ and in human eyes with glaucoma^40,41^.

Stiffening is further correlated with the maintenance of a round optic disc as stiffening particularly develops in a round pattern in the PPS region^18–21^. The orientation of the collagen and elastin fibrils is circumferential around the ONH in humans^42–44^. The arrangement predispose the region to strain/stress concentration in response to high IOP^19–21^, resulting in higher structural stiffness and thickness in the PPS^45^.

We questioned how the ONH could remain round in the congenital glaucomatous eye with a long axial length, that is, we queried how tilting of the optic disc was avoided. We considered that the strain-stiffening and circumferential tissue stiffening around the ONH may account for this in the round group. Anatomically, the progressive elongation of the ONH border tissue is an important component of the tilted optic disc in axial myopia^4,46^. The stiffening effect in the posterior sclera, including the neural canal, may resist stretching and elongation of the border tissue of Elschnig, thus, preserving the internally oblique configuration. This also suggests that the round discs are the result of a biomechanical mechanism, not random variance.

The aforementioned explanation is only applicable to the older group. From this study, we could not elucidate why the age at first surgery accounts for the two types of scleral response. We speculated that considering the rapid scleral growth in the first 6 months of life, achieving half of the total lifetime growth^47^, the relative degree of immaturity could be a factor. A significantly greater elastic extension in the younger group eyes may outweigh the component of stiffening, while in the older group eyes, the stiffening may outweigh the elongation. We focused on the BMO diameter measurements that would reflect a balloon-like “expansion” of the BM opening. The BMO diameter in the younger group was approximately 1.3 times greater than that in the older group, with values comparable to the normal values^48,49^. Moreover, the horizontal:vertical ratio did not change, suggesting an expansion from general eyeball enlargement. Significant thinning of the BM in myopic congenital glaucomatous eyes compared to the normal myopic eyes supports this speculation^24^.

It is also uncertain whether the tilting process in the younger eyes would be the same as that in the normal myopic eyes without an increased IOP. In myopia, the misalignment of the sclera, choroid, and retinal pigment epithlium-BM layers^50,51^ accounts for the tilted appearance. Shifting of the layered ONH structures has also been demonstrated^46,52^. The sclera in myopia does not simply slide, but it shows progressive elongation of the border tissue and a transition from internally to externally oblique configuration^4,46^. In this study, the ONH OCT images of the tilted discs appeared similar to those of the normal myopic eyes. The disc tilt progressed with axial growth as it did in normal myopia, suggesting a shared mechanism of scleral ECM remodeling^53^. Therefore, this mechanism should be evaluated in further studies.

**Figure 5:**
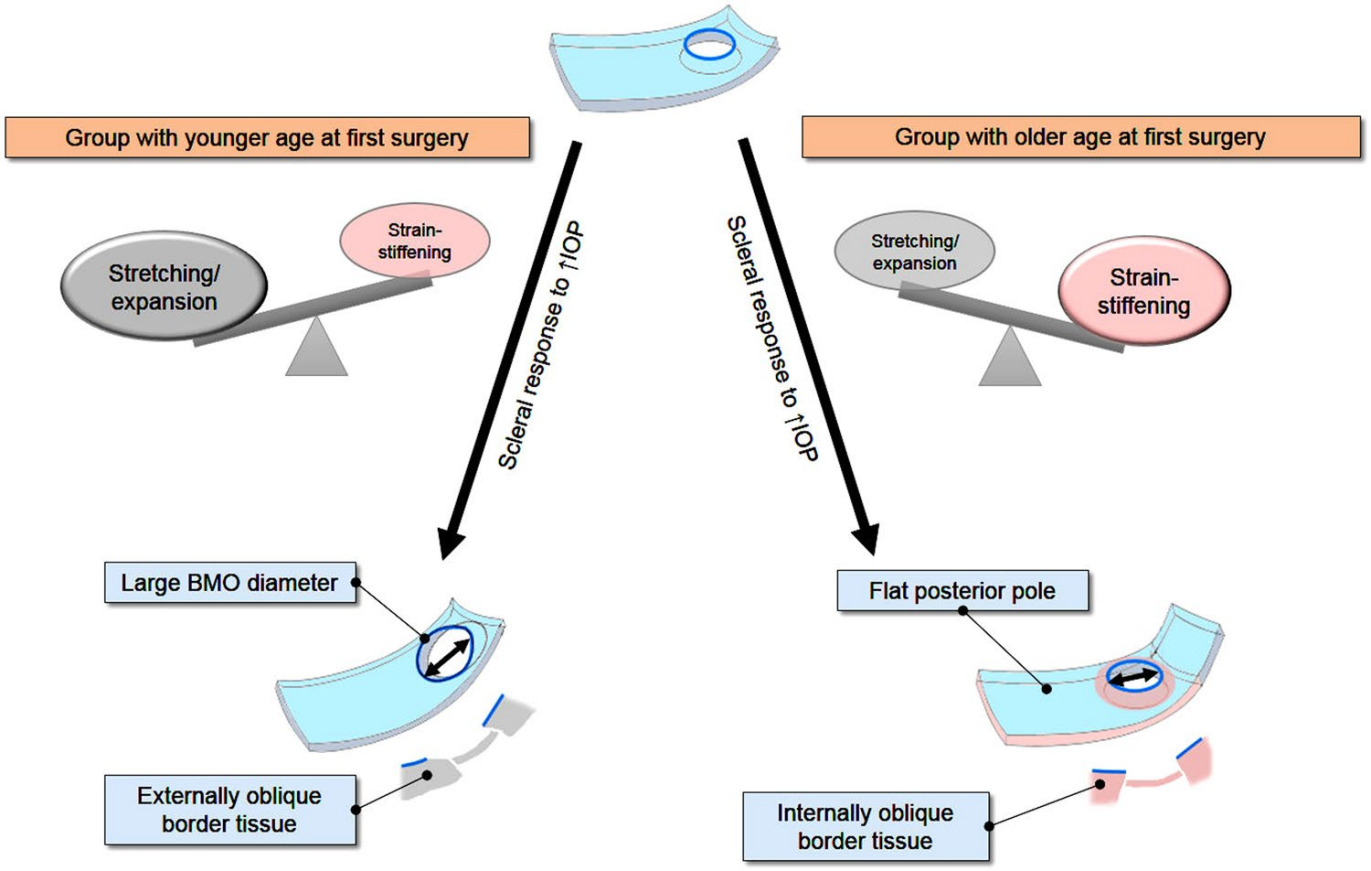
Hypothetical preservation of ONH roundness from the stiffening of PPS in response to the periods of elevated IOP in young eyes. Based on the evidenced biomechanical properties of the sclera to elevated IOP, either predominant stiffening of the peripapillary sclera (PPS) or predominant stretching/deformation may demonstrate as round or non-round optic disc shape, respectively. The stress concentration at the PPS, subsequent stiffening and resistance to deformation of the PPS in response to elevated IOP may be also applied to the eyes of the congenital glaucoma patients. It explains the internally oblique border tissue configuration that persists through progressive axial growth. In contrast, the significantly enlarged BMO diameter suggests more elongation of the border tissue through stretching deformation of the relatively more elastic sclera in the younger group than in the older group.

Our study results suggest that the posterior scleral changes parallel neither the corneal enlargement nor the cup reversal. As the posterior sclera stiffens, especially the circumferential PPS around the neural canal opening, the ONH and posterior pole may be spared from the predominant stretching in the older group (Fig. 5, right column). In the younger eyes, the response seems to be primarily elongation (Fig. 5, left column), but the specific reason remains unknown.

In the literature, Park *et al*.^11^ showed eyes of childhood glaucoma patients showed lesser change in the disc morphology during myopic shift than eyes with a normal disc. While our results are consistent with theirs, we also provide the OCT images of the deep ONH structures. We demonstrated the internally oblique configuration of the border tissue in myopic round discs. Moreover, not only the ONH neural canal, but the entire posterior pole, including the macular area, maintained a flat emmetropia-like contour.

With respect to the timing of stiffening, we also consider that the eyes in round disc group had already undergone remodelling of the scleral canal opening and PPS at the earliest documentation of optic disc photographs because the ovality index in newborns is around 1.2–1.3^54–57^, which is more oval than the early optic discs of our round group eyes. Moreover, the results of experimental IOP elevation provide evidence that scleral canal expansion occurs in a horizontal direction^28,30,58^, towards a round shape.

Throughout this investigation, we interpreted age at first surgery as the approximate representation of time of IOP elevation. However, an important additional factor that determines scleral response would be the duration of IOP increase^59^. We consider that roughly, the onset of IOP elevation and symptom presentation would be similar because congenital glaucoma is not an asymptomatic disease, but rather demonstrates vividly with corneal opacity, corneal enlargement, photophobia, and tearing in many cases^60^. In our hospital, the parents visited the hospital shortly after they had noticed abnormal signs. However, the exact onset of IOP elevation remains unknown, and a certain period of subclinical IOP elevation and symptomless cases are possible^60^. Therefore, it would be more prudent to state that the age at the first surgery may reliably reveal the timing when the degree and accumulated effect of IOP elevation have become significant enough to result in a detectable symptom, while the subclinical course remains speculative.

In terms of limitations of this study, we had a relatively small number of patients, and this was largely due to the rarity of the disease. However, the age difference was so distinct with little overlap that a larger number of patients is not likely to alter the significance. In addition, as this was a retrospective analysis, we could not examine the process of ONH deformation. Nevertheless, we had serial fundus photographs of the patients and observed that such patterning into either round or tilted was determined from the very early years. At least, we may reasonably assume the PPS outcome as a permanent structural sequel from the early period of elevated IOP before surgical control. Also, the distinct stiffening of the PPS should be investigated in patients, preferably using *in vivo* methods if possible. Despite the evidence supporting the stiffening of the PPS following elevation of IOP, a direct observation should be performed. Furthermore, the material properties, including the ECM composition before and after 6 months, should be investigated further. In addition, we acknowledge that the influence of fluctuating IOP on the scleral configuration and remodelling remains unevaluated. Nevertheless, notwithstanding the clinical difficulties, from the outcome of discrete bipartite grouping we considered that fluctuations of IOPs in the patients might not have affected the pattern of ocular growth as much as we suspected based on the various clinical courses. Besides, sampling IOP values within a period might also result in biased estimation of the actual cumulative IOP exposure in a given eye. Moreover, there was no difference in the number of surgeries or the age at last surgery, and most of the surgeries were terminated before age of five; about 40% only received a single surgery. For OCT image measurements, a concern regarding the ocular magnification factor due to the various posterior pole shapes in our myopic patient cohort could be raised. However, the issue does not interfere with our primary aim of investigation: whether the optic disc is round or not is determined regardless of the differences in length measurements. The numeric correlation between the roundness and ONH structural dimension, or the cumulative IOP elevations, were not the focus of this study. Lastly, the results can be applied specifically to eyes with episodes of elevated IOP during young ages, and not to normal axial myopic eyes.

We have not discussed the mechanism of scleral growth under normal IOP in depth. Increased creep rate predisposes the sclera to be more easily elongated over time while under a given IOP, even those within the normal range IOP, via altered viscoelastic properties of the sclera, such as thinning, decreased collagen fibril diameter, and altered collagen arrangement^61–63^ as a result of active remodelling. From our data, it seems that patients who received IOP insult at a young age might be left with permanent alterations in their scleral material properties and are now predisposed to ongoing axial length elongation. The related mechanisms should be investigated further.

In conclusion, age at first surgery was closely associated with the preservation of ONH roundness and internally oblique border tissue in surgically treated congenital glaucoma eyes; first surgery after 6 months of age in general was associated with a round ONH, while first surgery before that was associated with progressive optic disc tilting following axial growth. Considering the distinct period of IOP elevation, the results indicate a possible effect of the stiffening of PPS in the subset of congenital glaucoma eyes that are spared the progressive optic disc tilting seen in normal axial myopia. Future comprehensive imaging studies on the entire scleral shell shape are warranted.

## Methods

The medical records of patients with congenital glaucoma treated at Samsung Medical Center (Seoul, Korea) were retrospectively reviewed. This study was approved by the Samsung Medical Center Institutional Review Board, and the Institutional Review Board waived the requirement to obtain informed consent based on the retrospective nature of the study. This study adhered to the tenets of the Declaration of Helsinki.

### Inclusion/Exclusion

We included patients with primary congenital glaucoma and axial myopia. Diagnosis of congenital glaucoma was based on clinical findings before 36 months of age including elevated IOP, corneal opacity, oedema, increased corneal diameter, buphthalmos, and optic disc changes such as enlarged cupping. Patients with secondary glaucoma were excluded, such as Sturge-Weber syndrome (13 patients), aniridia (2 patients), oculodermal melanosis (3 patients), or previous congenital cataract surgery (16 patients).

Cut-off value of myopia was set at an axial length of 25.0 mm for clear contrast between tilted and non-tilted discs. As a cut-off value for optic disc tilt has not been established, we determined 25.0 mm to be appropriate considering the previous reports; the average axial length of eyes with tilted discs was 24.93 ± 1.1 mm in Singaporean myopic children^12^, and 25.52 mm in the Tanjong Pagar Study^1^. High myopia was defined as an axial length >26.0 mm^64–66^.

### Patient management and examinations

Surgeries were performed under general anaesthesia. As the primary surgery, goniotomy was performed for clear corneas, and trabeculotomy was performed for opaque or hazy corneas. In patients with repeated failed angle surgeries, trabeculectomy or combined trabeculotomy/trabeculectomy was considered, depending on the patient’s condition. Patients with repeated surgeries and uncontrolled IOP received glaucoma implant surgery. IOP-lowering eyedrops were used in the bridge period to control IOP. Patient visits were scheduled every 6 months in cases of stable IOP. In cases of uncontrolled IOP, more frequent follow-ups were recommended and interventions to lower the IOP were performed without delay.

Preoperative IOP measurement with a Perkins tonometer (Kowa HA-2, Kowa, Tokyo, Japan), and anterior segment examination with a portable slit-lamp were performed after sedation using chloral hydrate syrup. We performed regular postoperative examinations of refractive error, IOP, and axial length, and optic disc and fundus photography. For axial length measurements, an ultrasound biometer (Haag-Streit, Bern, Switzerland) was used under sedation, whereas partial coherence interferometry (IOLMaster, Carl Zeiss Meditec, Dublin, CA, USA) was preferentially used for patients older than 10 years or for those who could cooperate. A handheld portable fundus camera (Kowa Genesis, Kowa, Tokyo, Japan) was used for optic disc and fundus photography under sedation, while colour and red-free fundus photography (Topcon, Paramus, NJ, USA) were used for patients older than 10 years or for those providing adequate cooperation.

### Posterior pole and ONH evaluation using enhanced depth imaging OCT

Enhanced depth imaging OCT (Heidelberg Engineering, Heidelberg, Germany) was performed in two modes: 12 radial-line B-scans centred on the optic disc and a wide scan connecting the fovea to the centre of the optic disc (FoDi). In patients providing less cooperation, at least the horizontal and vertical sections on the ONH were acquired with some effort. Horizontal and vertical BMO diameters and LC depth were also measured. LC depth was measured as the perpendicular distance from the BMO plane at three points: at the centre of the LC and at two points 200 µm apart in the temporal and nasal directions.

**Figure 6:**
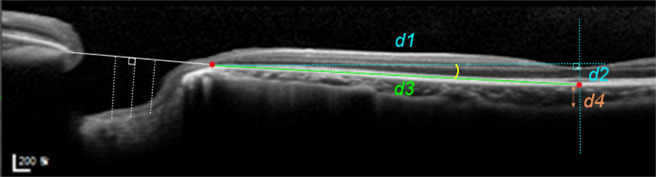
Measurement of the posterior pole and optic nerve head structures using enhanced depth imaging optical coherence tomography. To calculate the macular curvature, an imaginary right-angled triangle was set, with Bruch’s membrane opening (BMO) at the temporal disc margin and subfoveal Bruch’s membrane (BM) as the two apices (red dots). A line passing through the inner fovea and subfoveal BM was used as another side. Curvature of macular BM was estimated by the angle (yellow arc) from the arctangent of the triangle (d2/d1), where the hypotenuse was set to reflect macular BM length (d3). Horizontal BMO distance measurement was performed where shown by the white line, and lamina cribrosa depth was measured from the line at three points: at the centre and two lateral sides 200 μm away in the temporal and nasal directions (white dotted lines). Subfoveal choroidal thickness was measured on the perpendicular line (d4).

In the FoDi image, curvature of macular BM was calculated as the angle defined by the arctangent of an imaginary right-angled triangle that we set with the BMO at the temporal disc margin and subfoveal BM as the two apices. The hypotenuse was set to reflect macular BM length, and the line connecting the inner fovea and subfoveal BM was used as one side. Curvature was defined as the sharp angle from the arctangent value (Fig. 6). When the subfoveal point was positioned above the temporal BMO, the distance was recorded as a negative value. Subfoveal choroidal thickness, scleral border tissue length and zone β length were also measured. Measurements were performed using a built-in software, and the scaling of the OCT scanner was adjusted to 1:1 μm before measurement.

### Patient grouping for analysis

**Figure 7:**
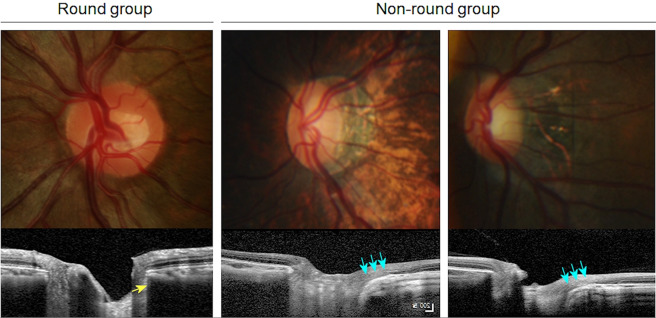
Grouping of the round and non-round groups. The optic discs in myopic congenital glaucoma patients were grouped into round and non-round groups. The round group was defined as having an ovality index under 1.1 and preserved structural configuration in the peripapillary sclera–scleral border tissue–lamina cribrosa complex, where border tissue was internally oblique (yellow arrow) or minimal with a measured length <100 μm on optical coherence tomography. In contrast, the non-round group included various types of tilted discs and externally oblique border tissue (blue arrows).

Patients were divided into groups with (round group) and without (non-round group) preserved roundness of the ONH (Fig. 7). The round group was defined as having preserved structural configuration in the PPS–scleral border tissue–LC complex. We included only the eyes that met both of the following criteria: (1) an ovality index, defined as the ratio of longest and shortest diameter of clinical optic disc margin, of <1.1 on colour photographs; and (2) an internally oblique, nonoblique, or minimally externally oblique scleral border tissue <100 μm on OCT. The non-round group included various types of tilted discs.

For statistical analysis, we used a generalised estimating equation approach for comparison between the non-round and round groups, considering the possible correlation between the eyes of the same patient^67^. Sex, age at the first surgery, preoperative IOP, axial length, number of surgeries, and age at the last surgery, as well as OCT parameters, were compared between the groups. Axial length values measured at the most recent visit within 6 months of the day OCT was performed were used for analysis. Multiple logistic regression was performed by including only parameters that were clinically meaningful as causative factors, using axial length values obtained at around 10 years of age to consider the age-related growth. All statistical analyses were performed using IBM SPSS software version 22.0 (SPSS, Inc., Chicago, IL, USA). A P value less than 0.05 was considered statistically significant.
